# Metal‐Less Top Electrode Semitransparent Organic Solar Modules with an Average Visible Transmission of 51% and a Light Utilization Efficiency of 4%

**DOI:** 10.1002/advs.202507521

**Published:** 2025-06-23

**Authors:** Leonie Pap, Mathias List, René Haberstroh, Lasse Bienkowski, Martin Mattenheimer, Thomas Kroyer, Jared Faisst, Birger Zimmermann, Uli Würfel

**Affiliations:** ^1^ Fraunhofer Institute for Solar Energy Systems ISE Heidenhofstr. 2 79110 Freiburg Germany; ^2^ Materials Research Center FMF University of Freiburg Stefan‐Meier‐Straße 21 79104 Freiburg im Breisgau Germany; ^3^ Department of Ion Physics and Applied Physics University Innsbruck Technikerstraße 25 Innsbruck 6020 Austria

**Keywords:** high average visible transmission, light utilization efficiency, optical modelling, reflecting back electrode, semi‐transparent organic solar modules

## Abstract

Semitransparent organic modules (STOMs) are of particular interest due to potential applications in building‐integrated photovoltaics though upscaling without significant performance losses while ensuring an aesthetic appearance for window applications remains a major research obstacle. This study presents two types of STOMs with an area of 11.4 cm^2^, exhibiting homogeneous appearance while retaining up to 92% of their respective small‐area cell power conversion efficiency (PCE). Precise laser structuring minimized the area loss originating from the sub‐cell series interconnection, allowing narrow cell stripes down to 1.25 mm. This enables a metal‐less top electrode composed solely of PEDOT:PSS without compromising fill factor. For a module, an additional challenge is to ensure a low resistance series interconnection between sub‐cells. This is realized with a novel approach based on a direct contact between PEDOT:PSS and a thin Ag layer within the back electrode. For the first design, the latter incorporates Al‐doped ZnO and Ag attaining a PCE of 6.1% with an average visible transmission (AVT) of 47.5%. A more elaborate design features an extended back electrode using TiO_2_ and SiO_2_ for improved optical performance (PCE = 7.9%), reaching an unprecedented AVT of 50.8% and a light utilization efficiency (LUE = AVT x PCE) of 4.0%.

## Graphical abstract

Semitransparent organic modules with device areas of 11.4 cm^2^ are developed for building‐integrated photovoltaics. Including a laser‐structured, metal‐free top electrode, the module achieves a PCE of 6.1% with an AVT of 47.5%. An extended back‐electrode design enhances optical and electrical performance, achieving PCE of 7.9%, AVT of 50.8%, and LUE of 4.0%, demonstrating scalable, aesthetic solutions for energy‐efficient window applications.

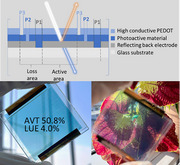

## 1 Introduction

The increasing demand for sustainable energy solutions has driven significant interest in semi‐transparent organic solar cells (STOSCs), particularly their potential to integrate seamlessly into windows and building facades.^[^ ^1^, ^2^, ^3^, ^4^, ^5^ ^]^ These photovoltaic devices are capable of generating electricity while maintaining partial transparency in the visible range, making them highly suitable for urban environments where space for sustainable energy generation is limited.^[^ ^6^ ^]^ This dual functionality is particularly advantageous in densely populated areas, where maximizing the utility of building surfaces is a valuable advantage for sustainable development.^[^ ^7^, ^8^, ^9^ ^]^

Unlike their inorganic counterparts, organic semiconductors offer the distinct benefit of tunable optical properties via molecular design. By adjusting the molecular structure of the organic semiconductor, it is possible to selectively absorb light in the near infrared region while maintaining transparency in the visible part of the spectrum.^[^ ^10^, ^11^, ^12^, ^13^, ^14^, ^15^, ^16^, ^17^ ^]^ This ability to modulate absorption characteristics enables STOSCs to harvest solar energy efficiently without compromising the aesthetic or functional requirements of window applications. Consequently, these materials represent a promising solution for integrating energy generation into transparent surfaces, such as windows, without obstructing visible light.

On the single‐cell scale, semi‐transparent organic photovoltaics in literature have already successfully reached impressive light utilization efficiencies (LUE  = AVT x PCE) of over 5.0%. Chen et al. reported a molecular weight‐regulated sequential deposition strategy to attain a favourable morphology, reaching a PCE of 10.01% with an impressive AVT of 50.05%, resulting in a LUE of 5.01%.^[^ ^18^ ^]^ While a color rendering index (CRI) is not mentioned to demonstrate color neutrality, the Commission Internationale d'eclairage (CIE) chromaticity coordinates for this cell are given as x,y (0.265, 0.290). An LUE of 3.5% was reached by Forrest et al. employing an antireflection coating and outcoupling layers, resulting in an AVT of 43.3%, a PCE of 8.1%, and a CRI of 86.^[^ ^19^ ^]^ Two record LUE values were achieved by Gang Li et al., who reported a LUE of 5.35% and CRI of 85.39 using an aperiodic band‐pass filter electrode, and Yan‐Qing Li et al., who reached a LUE of 5.6% and CRI of 87.8 using a similar principle, including ZnS and MgF_2_ layers for optical enhancement of the silver electrode.^[^ ^20^, ^21^ ^]^

For STOSCs to transition from laboratory‐scale research to real‐world implementation, the challenge of upscaling must be addressed. Large‐area production requires maintaining surface homogeneity for aesthetic appeal without compromising power conversion efficiency (PCE). Uniformity in transparency is crucial for window applications, where the appearance of the photovoltaic layer must complement architectural design while delivering efficient energy generation. However, when scaling up from small cell areas to larger module sizes, reductions in efficiency and fill factor (*FF*) often occur due to increased series resistance (*R*_S_), defects, and non‐uniformities in the thin‐film layers.^[^ ^1^, ^22^ ^]^ Additionally, large‐scale fabrication processes must meet industry standards, such as avoiding the use of halogenated solvents.^[^ ^23^ ^]^ Balancing these various factors presents a complex and demanding task in this field of research.

Recent advancements in the development of semi‐transparent organic modules (STOMs) have shown promising potential. To minimize efficiency losses caused by interconnections, different technologies have been adopted in organic photovoltaics. These new technologies aim to enable interconnections in the micrometer scale, ensuring a high geometric fill factor (*GFF*), which is defined as the ratio between the photovoltaic active area and the total module area. Logothedtidis et al. presented the implementation of a laser ablation patterning process using ultrafast picosecond pulses in the manufacturing of fully roll‐to‐roll printed flexible semitransparent OPV modules with a 3.4% power conversion efficiency, 91% *GFF*, and 29% maximum transmittance at 504 nm.^[^ ^24^ ^]^ In an alternative approach, Forrest et al. demonstrated a multilevel peel‐off patterning method to achieve a *GFF* of 95.8% for a STOM reaching PCE of 7.3% and AVT of 41.8%, resulting in a light utilization efficiency of 3.1%.^[^ ^25^ ^]^ While these and several other existing state‐of‐the‐art devices demonstrate impressive developments in the field, few STOMs show AVT values exceeding 50% and LUEs of over 3.5%. Achieving large‐area modules with high visible transmission, uniformity, high *GFF*, and minimal *FF* losses remains a significant technical hurdle.

In this work, we introduce two innovative indium tin oxide (ITO)‐free STOMs, each with a device area of 11.4 cm^2^, that deliver a visually homogeneous appearance while reaching a *GFF* of up to 96.8%. Our strategy effectively combines advanced technologies to create highly transparent, scalable, solution‐processed STOMs, all fabricated with halogen‐free solvents. These designs not only ensure a uniform surface but also enable seamless integration into window applications without compromising performance. The first design based on previous work features a multilayered back electrode composed of aluminum‐doped zinc oxide and silver, paired with a metal‐free top electrode comprising a highly conductive poly(3,4‐ethylenedioxythiophene) polystyrene sulfonate (PEDOT:PSS) formulation from Heraeus Clevios, referred to as SCA2003.^[^ ^26^ ^]^ A key innovation in this design is the low‐resistive series interconnection between adjacent sub‐cells through a direct contact between the metal‐less top electrode (i.e., PEDOT:PSS) with a thin Ag layer in the back electrode. This is achieved by precise and selective laser ablation. Additionally, the narrow laser patterning minimizes interconnection area losses. This enables the production of two different types of modules featuring either 12 (module type 1) or 24 (module type 2) sub‐cells with reduced cell stripe widths of 2.5 and 1.25 mm, respectively. As a result, *R*_S_ losses are minimized without significant reductions in *GFF*. This architecture achieves a power conversion efficiency (PCE) of 6.1% and an average visible transmission (AVT) of 47.5%, demonstrating both high efficiency and transparency. The second design goes even further by incorporating an optically optimized back electrode using SiO_2_ and TiO_2_, as well as an additional ZnO electron transport layer (ETL) significantly enhancing both optical and electrical performance, as demonstrated on a small area cell level in our previous work.^[^ ^27^ ^]^ This design reaches a PCE of 7.9% and a remarkable AVT of 50.8%, leading to a module light utilization efficiency of 4.0% which to the best of our knowledge, is the highest value for a semitransparent organic module. Maintaining up to 92% of the performance of their smaller counterparts this breakthrough represents a significant step forward in achieving the ideal balance between transparency, scalability, and energy generation for building integrated photovoltaics.

## 2 Results and Discussion

**Figure 1:**
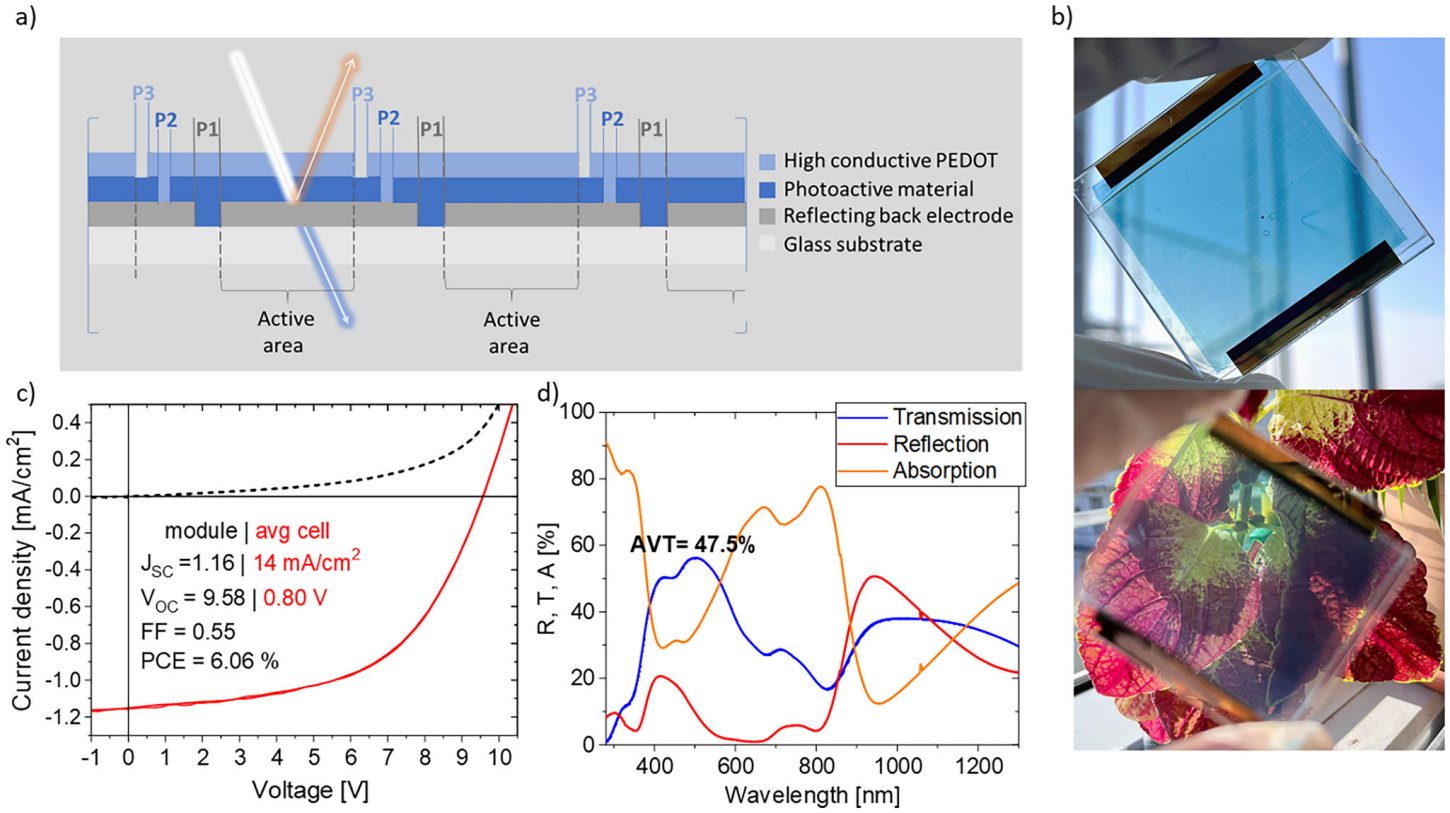
a) Schematic representation of the module architecture, highlighting the interconnection points and laser structuring lines P1‐P3. An additional P4 line is included on the sides of the module to define the width of the active area. The infrared part of incoming light is reflected by the back electrode, enabling additional absorption in the photoactive layer, while visible light is transmitted. b) Photograph of an encapsulated module in front of the sky (above) and a colorful plant (below). c) JV‐curve of the AZO|Ag|AZO|PV‐X Plus|HTL‐X|SCA2003 module (referred to as type 1) with a photoactive layer of 60 nm, including full module (12 cells) performance parameters as well as average performance parameters calculated per cell within the module. d) Transmission, reflection, and absorption spectra of the complete layer stack.

In our previous work, two distinct cell architectures were developed, each including an infrared mirror as a back electrode to enhance both optical and electrical performance.^[^ ^26^, ^27^ ^]^ The first configuration used a multilayer back electrode composed of aluminum‐doped zinc oxide and silver (AZO|Ag|AZO), as well as a photoactive layer consisting of PV‐X Plus from RaynergyTek (see experimental section in Supporting Information). Additionally, two layers of PEDOT:PSS, labeled HTL‐X and SCA2003, are deposited on top. The HTL‐X layer promotes proper electrical alignment and wetting for the subsequent SCA2003 layer, which in turn provides sufficient conductivity. This solar cell achieved an average visible transmission (AVT) of 46.3% and a power conversion efficiency (PCE) of 8.7%. The light utilization efficiency (LUE) of this device reached 4.0%.^[^ ^26^ ^]^ To advance from laboratory‐scale research to practical applications, scaling up fabrication is a crucial next step. Accordingly, a mini‐module layout (referred to as type 1) was designed, connecting 12 individual cells in series with a cell stripe width of 2.5 mm (including an interconnection loss width of about 80 µm), resulting in a total module area of 11.4 cm^2^ (**Figure** **1**b). To achieve a high *GFF*, minimizing the interconnection area between the individual cells is essential. For this purpose, monolithic laser ablation is applied to precisely structure the layers at specific stages of the fabrication process (Figure 1b). The top electrode of the device is composed of a high‐conductive poly(3,4‐ethylenedioxythiophene) polystyrene sulfonate (PEDOT:PSS) layer, referred to as SCA2003, which was leveraged to establish direct contact to the silver layer within the back electrode. To ensure the selective ablation of the appropriate layers within the stack, careful optimization of laser energy and pitch distance was performed. A picosecond UV laser with a wavelength of λ = 343 nm was used. This approach enabled the precise patterning required for effective module integration with a PCE of 6.1% and a *GFF* of 96.8%.

To achieve a higher AVT, the thickness of the PV‐X Plus layer was slightly reduced (from 66 to 60 nm) compared to the best‐performing single cell from our previous work. To accurately assess the performance values per cell within the module type 1 against those of the upscaled module relative to a single cell, the following table (**Table** **1**) compares the calculated average performance measured and simulated for a single cell with identical layer thicknesses and architecture. This allows for a direct evaluation of the module's efficiency compared to its single cell counterpart.

**Table 1:** Calculated average parameters per cell within the module type 1 compared to measured and simulated parameters of a single cell with comparable layer thicknesses and identical architecture.

	Calculated average performance of cell within module		Average measured performance of single cell (total of 6 cells)	Optical simulations of single cell
	total module area	active area: corrected for *GFF* losses		
*J*_sc_ [mA cm^−2^]	13.9 | 14.4		15.0 ± 0.37	15.1
*V*_OC_ [V]	0.80		0.82	–
*FF* [1]	0.55		0.65	–
PCE [%]	6.1 | 6.3		7.9 ± 0.60	–
AVT [%]	47.5		47.2	47.6
LUE [%]	2.9		3.7	–

The average short‐circuit current density (*J*_SC_) of the sub‐cells is slightly lower with 13.9 mA cm^−^^2^, compared to 15.0 mA cm^−^^2^ for the single cell average. When only taking into account the active area of a sub‐cell (i.e., without the *GFF* loss due to the interconnection), the average sub‐cell *J*_SC_ increases to 14.4 mA cm^−2^. Losses in fill factor (*FF*) and open‐circuit voltage (*V*_OC_) are likely due to increased internal resistance or less efficient charge extraction in the module structure, as indicated by the slope of the module JV‐curve at −1 V. Consequently, the power conversion efficiency (PCE) of the module also decreases, from 7.9% in the single cell to 6.1% in the module, representing a notable drop in energy conversion efficiency during the transition from a single cell to a module. Despite these declines in electrical performance, the AVT remains almost unchanged, with the module achieving 47.5% compared to 47.2% for the single cell. This suggests that the optical properties were successfully maintained throughout the adjustments in layer thicknesses for both devices, demonstrating promising scalability. This represents a relatively straightforward process of upscaling to a module with a metal‐free top electrode, solution‐processed using halogen‐free solvents, which successfully integrates multiple cells in series while preserving reasonable electrical performance, and aesthetic uniformity with a high *GFF* of 96.8%.

**Figure 2:**
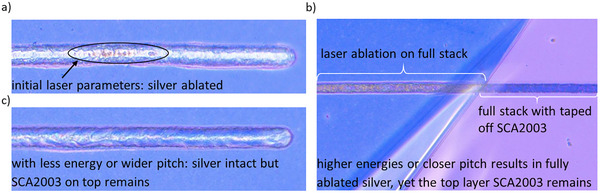
UV ps laser patterning of the module (type 2) displaying microscope pictures of a) initial P3 laser parameters of 0.085 µJ and 3 µm pitch and b) laser ablation of the full stack with higher energies/closer pitch. On one side of the full stack, the top PEDOT SCA2003 was taped off after laser structuring. The taped‐off side reveals, that despite the laser´s higher energies/closer pitch, SCA2003 remains on top of the ablated line. c) laser ablated structures on full module stack with less energy/wider pitch. The silver electrode remains intact, however the top SCA2003 remains as well.

Rather than further optimizing this module, it is instead used as proof of concept to demonstrate feasibility, with efforts redirected toward improving efficiency by implementing this approach in the upscaling of a different cell design that has exhibited the best overall performance.^[^ ^27^ ^]^ This single cell consists of an optically extended back electrode that incorporates additional SiO_2_ and TiO_2_ layers for better optical performance as well as an additional ZnO electron transport layer for improved electrical performance (Full stack: TiO_2_|SiO_2_|TiO_2_|AZO|Ag|AZO|ZnO|PV‐X Plus|HTL‐X|SCA2003).^[^ ^27^ ^]^ The new architecture will be referred to as type 2. Initial experiments to incorporate this layer stack into the module design used for the single silver device yielded poor outcomes, with performances severely limited by shunting, resulting in *FF*s below 30%. To investigate the cause of this issue, the laser ablation structures were inspected with a microscope (VHX‐6000) (**Figure** **2**). Note that the identification of the different layer combinations is based on the respective colors (caused by interferences) in which each of these layer stacks appear under the microscope. These colors were validated by investigating known layer combinations. Among all laser‐structured lines, the P2 and P3 lines represent the most critical processes, as it is essential to selectively ablate the layer stack down to the silver layer within the back electrode, or in the case of the P3 line, successfully remove the SCA2003 top layer. Insufficient ablation, which – in the case of the P2 line – fails to adequately expose the silver layer, results in poor electrical contact between the top and bottom electrode, hindering cell interconnection within the module. Conversely, excessive ablation may damage or completely remove the silver layer, leaving no material for the top contact to effectively connect with, thereby compromising the module's functionality.

Generally, two laser systems were used, a UV picosecond laser (λ = 343 nm) and an IR femtosecond laser (λ = 1030 nm). Selective ablation until the silver layer within the back electrode for the P2 was enabled by using the UV laser with a fluence of 0.034 J cm^−2^ and a laser beam radius of 4.35 µm. However, when trying to find the right parameters for the P3 line for the stack that now includes a top layer of SCA2003, the following observations were made: When ablating with an energy of 0.085 µJ and a 3 µm pitch, microscope images reveal a laser‐structured line that appears to have ablated all top layers down to the silver electrode (Figure 2a). Additionally, in some regions of the laser‐structured line, partial ablation of the silver is observed, exposing the underlying layers. These areas potentially contribute to the poor performance of the modules. At higher energies and closer pitch, the silver layer was fully ablated (Figure 2b). Interestingly, it was also observed that taping off the SCA2003 layer from the full stack resulted in a change in the color of the laser lines, suggesting that despite the ablation of the silver layer, the top SCA2003 layer remains intact. Similarly, at lower energies and wider pitch, the silver layer remained intact, however, the SCA2003 layer was visibly still present as well (Figure 2c).

**Figure 3:**
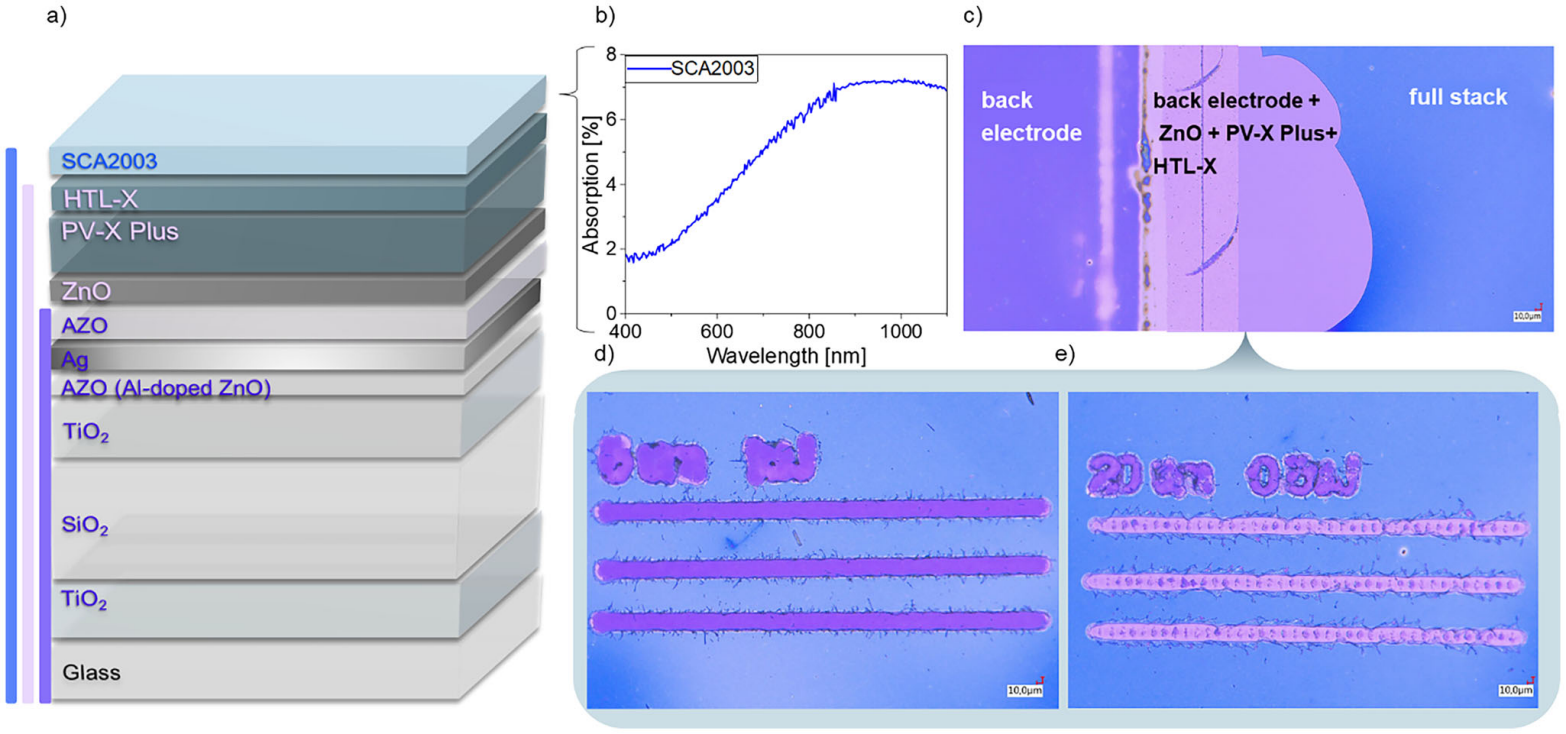
a) Schematic depiction of the full module type 2 layer stack, including reference for colors of each layer sequence under the microscope b) Absorption spectrum of a SCA2003 layer, c) microscope picture of the electrode (violet), full stack minus SCA2003 (pink) and full stack (blue) for color reference. Selective laser structuring down to d) the electrode with the IR fs laser at an energy of 1 µJ and 5 µm pitch and e) HTL‐X at an energy of 0.5 µJ and 20 µm pitch. With a laser beam radius of 13 µm, these energies correspond to fluences of 0.34 and 0.17 J cm^−2^, respectively.

To investigate optimal conditions for accurately ablating the SCA2003 layer, the absorption spectrum was measured (**Figure** **3**b). Results showed that while the PEDOT:PSS generally exhibits high transparency, absorption increases notably in the infrared range. To ablate the SCA2003 more effectively – with reduced energy input and thus minimized risk of damage to the silver layer within the bottom electrode – the previously used UV laser was substituted by the IR laser for the P3 line. After adjusting the laser parameters, particularly energy and pitch, it was possible to selectively ablate all layers down to the electrode (Figure 3d) or even just the SCA2003 itself (Figure 3e) without damaging the silver layer.

**Figure 4:**
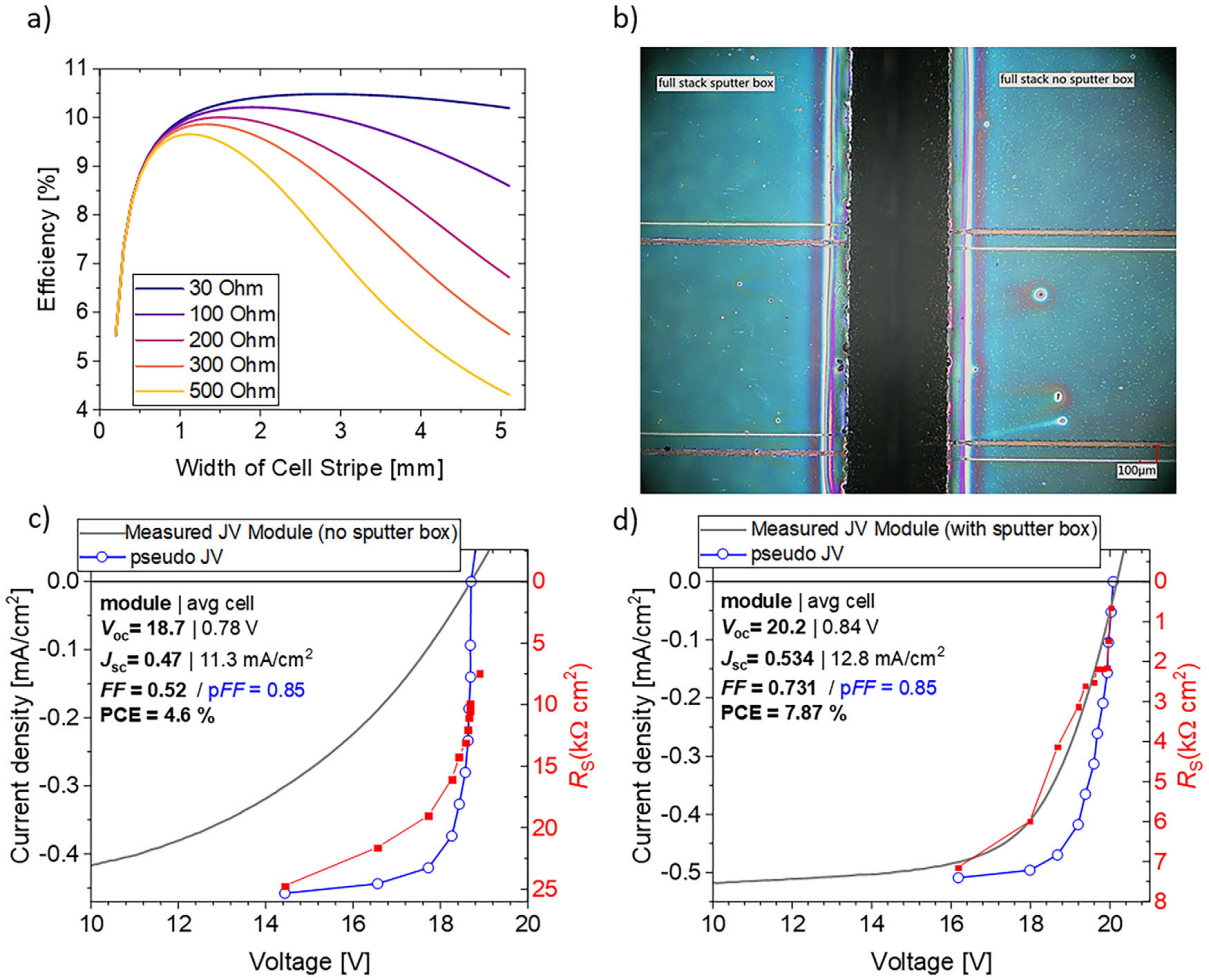
a) Calculated efficiencies dependent on cell stripe width and sheet resistance of the top contact material SCA2003. b) Microscope picture of part of the full module stack with (left) and without (right) the usage of the protective sputter box. JV‐curve (grey), Suns‐*V*_OC_ measurement (blue) and calculated series resistance (*R*_S_) (red) of the module type 2 with 24 interconnected cells c) without and d) with a protective enclosure.

Additionally, several processing steps were optimized to minimize exposure to ambient conditions and reduce the time intervals between each fabrication stage. Next, the optimal cell stripe width was calculated considering two limitations, the loss in active area due to the sub‐cell interconnection on the one hand, and *FF* losses due to the sheet resistance of the SCA2003 top electrode on the other hand. Details of the calculation model and parameters can be found in the experimental section in Supporting Information. The active area loss originating from the interconnections of the sub‐cells is about 100 µm, and the sheet resistance of SCA2003 is ≈300 Ω for the used thickness of 70 nm. **Figure** **4**a shows the results of these numerical simulations from which we extracted an ideal cell stripe width of ≈1.25 mm. Note that this rather small value is enabled by the minimal area loss related to the laser structuring. This, in turn is very favorable and allows achieving good fill factors despite a rather limited conductivity of a metal‐less top electrode comprising solely PEDOT:PSS. Another significant improvement was achieved by installing a protective enclosure within the sputtering machine, effectively shielding the glass substrates from dust particles during the evacuation process (Figure 4b).

Figure 4c) and d) depict the JV‐curves of the modules with 24 interconnected cells built according to the type 2 layer stack. While the module related to Figure 4c) was processed without the enclosure, the other module (Figure 4d) incorporated this protective measure during the evacuation process of the sputtering machine to enhance the electrode quality. External quantum efficiency data of the latter can be found in Figure S2, Supporting Information. The results reveal substantial performance improvements for the module sputtered with the enclosure, with the *V*_OC_ increasing from 18.7 V to 20.1 V. Additionally, the *J*_SC_ improved from 0.47 mA cm^−^^2^ in the unprotected module to 0.52 mA cm^−^^2^ in the module with the enclosure. The most significant improvement however is seen in the *ff*, rising from 0.52 for the unprotected module to 0.73 for the one with enclosure. This boost in *FF* can be explained with a combination of an increased parallel resistance (*R*_p_) and a decreased series resistance (*R*_s_) as will be outlined in the following. First, to determine the effect of *R*_S_ related losses, light intensity dependent open‐circuit voltage measurements (Suns‐*V*_OC_) were conducted to reconstruct a pseudo JV curve that is free of the influence of the series resistance (see experimental section in Supporting Information). The pseudo JV curves are depicted as blue lines with symbols in Figure 4b,c). Notably, the *pFF* (pseudo *FF*) remains consistent at 0.85 for both modules, indicating that the intrinsic electronic properties were unaffected by the protective enclosure. The values of *R*_S_ at each current can be calculated according to *R*_S_ = Δ*V*/*J* wherein Δ *v* is the difference in voltage of the normal and the pseudo JV‐curve, respectively.^[^ ^28^ ^]^ The *R*_S_ values are indicated in red in Figure 4b,c). Both modules show a reduction in *R*_S_ with increasing light intensities (corresponding to higher voltages), attributable to an increase in charge carrier concentration, which in turn improves the conductivity.^[^ ^28^ ^]^ Notably, the module with the sputter box shows *R*_S_ values that are almost an order of magnitude lower than the module without the sputter box, demonstrating a substantial improvement that contributes to the increased fill factor.

**Figure 5:**
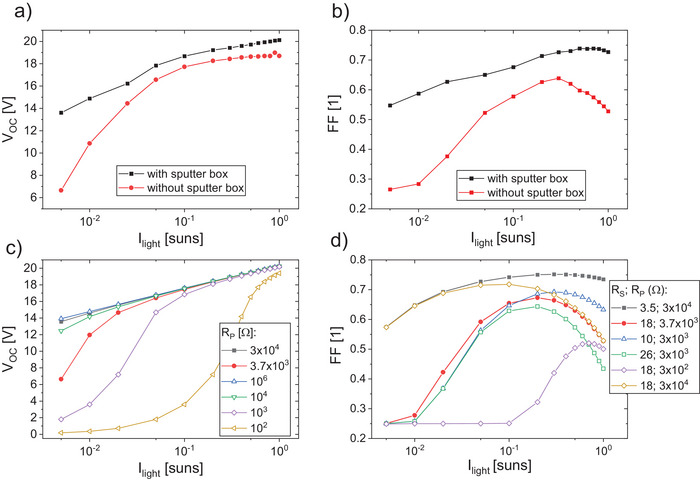
a) measured *V*_OC_ and b) *FF* as a function of light intensity for the module with (black) and without (red) sputter box, c) simulated *V*_OC_ for different values of the parallel resistance *R*_P_, and *FF* as a function of light intensity for different values of series resistance *R*_S_ and parallel resistance *R*_P_.

Additionally, the measured JV‐curve of the module without the sputter box shows a much stronger voltage drop for lower light intensities (corresponding to lower voltages) relative to the module with the sputter box. To gain more insight into this phenomenon, the measured *V*_OC_ and *FF* were plotted as a function of the respective light intensity (**Figure** **5**).

Figure 5a,b demonstrates the previously mentioned trend more clearly. The module with the sputter box exhibits an overall higher *V*_OC_ as well as a more linear decrease toward lower light intensities compared to the module without the sputter box. For the latter, a breakdown toward lower intensities of the *FF* at roughly 0.3 suns and of *V*_OC_ at about 0.1 suns is observed. This is a clear sign of the detrimental impact of a low parallel resistance. In addition, the *FF* also shows a significant reduction from 0.3 suns toward higher intensities for the module without the sputter box, whereas for the one with enclosure, there is only a very slight reduction when approaching an intensity of 1 sun. This can be attributed to the already discussed difference in *R*_S_ in a straightforward manner. To support this analysis, we have carried out numerical simulations based on the analytical two‐diode model including a series and a parallel resistance which can be described by the extended Shockley diode equation:

(1) $$\begin{array}{cc} J\left(V\right) & =J_{01}\left[\exp\left(\frac{e_{0}\left(V-JR_{S}\right)}{k_{B}T}\right)-1\right]\\ & \;+J_{02}\left[\exp\left(\frac{e_{0}\left(V-JR_{S}\right)}{2\;k_{B}T}\right)-1\right]\\ & \;+\frac{V-JR_{S}}{R_{P}}-J_{\mathrm{Gen}} \end{array}$$

Therein, *J*_01_ and *J*_02_ are the saturation current densities of the two diodes with ideality factors of 1 and 2, e_0_ the elementary charge, *k*_B_ the Boltzmann constant, *T* the temperature and *J*_Gen_ the photogenerated current density. Note that we do not expect to be able to exactly reproduce the experimental data with this zero‐dimensional model but rather intend to work out the intensity‐dependent, archetypical behavior in the presence of series and shunt resistance. Note that in this model, the value of *V*_OC_ is independent of the value of *R*_S_ as there is zero current flow at open‐circuit conditions. It can be seen in Figure 5c that there is a rather clear breakdown of the open‐circuit voltage at a certain light intensity (toward lower light intensities) and the value of the latter depends sensitively on the value of *R*_P_. The lower the value of *R*_P_, the sooner the breakdown occurs. From this, it can be derived that if in a module only a small number of cells is (severely) shunted, one will expect its *V*_OC_ (as function of light intensity) to show a step‐like behavior as shunted sub‐cells do (almost) not contribute to the voltage anymore. The fact that this is not observed (see Figure 5a), especially not for the unprotected module, indicates that many (if not all) sub cells in the module are actually affected by local shunts. This hypothesis is strongly confirmed by the microscopic images in Figure 4a) revealing a large number of small spots. We find that the values of *R*_P_ = 3×10^4^ Ωcm^2^ and *R*_P_ = 3.7×10^3^ Ωcm^2^ (black and red filled symbols in Figure 5c) reproduce the experimental data of the protected and unprotected module in a reasonable manner.

Toward higher intensities, the impact of *R*_P_ on both, *FF* and *V*_OC_, decreases. The experimentally observed significant *FF* decrease of the unprotected module between about 0.3 and 1 sun can be assigned to the larger *R*_S_, as derived from the voltage difference between normal and pseudo JV‐curve, Figure 4c. In Figure 5d, the parameter combination *R*_S_ = 18 Ωcm^2^ and *R*_P_ = 3.7×10^3^ Ωcm^2^, reproduces this behavior, that is, the decrease of *FF* and *V*_OC_ toward lower intensities and the decreasing *FF* toward higher intensities of the unprotected module, rather well. For the module sputtered with the enclosure, *R*_S_ = 3.5 Ωcm^2^ and *R*_P_ = 3.7×10^4^ Ωcm^2^ approximate the experimental data with reasonable accuracy. Further, Figure 5d also shows results for other parameter combinations. It can be seen that a smaller value for *R*_S_ reduces the decrease in *FF* at higher light intensities whereas a smaller value for *R*_P_ increases the impact toward lower light intensities. Note again that the 0D two‐diode model is of course not capable to capture any spatial inhomogeneity that for sure play a role here. Nevertheless, it helps to understand the fundamental correlations between series and parallel resistance and the illumination intensity dependence of open‐circuit voltage and fill factor. And a more complex (e.g., 2D) simulative approach is beyond the scope of this work that is focused on experimental investigations of how to achieve optimized performance of transparent OPV mini‐modules. Finally, the experimentally observed saturation of the open‐circuit voltage of the unprotected module at high intensities (Figure 5a) can be explained with surface recombination as its impact on *V*_OC_ becomes more pronounced with increasing light intensity. Beyond doubt, the most important finding here is that protecting the module from the exposure to dust particles during the sputtering process is essential and leads to clear improvements in performance due to a reduced series resistance and the reduction of local shunts.

Consequently, the power conversion efficiency (PCE) improved from 4.6% without the enclosure to 7.9% with it, reflecting the cumulative gains in *V*_OC_, *J*_SC_, and *FF*. In summary, these findings indicate that the use of a protective enclosure during sputtering significantly enhances module performance by reducing particle contamination. This improvement results in an overall maximum performance of 7.9% PCE with a significant AVT of 50.8%, achieving an overall LUE of 4.0% (Figure 4d). The overall *GFF* of this module is 92.3%. To ensure an accurate assessment of the performance, the calculated average performance of a cell within the module is compared to the average measured performance and optical simulations for a single cell with comparable layer thickness and architecture (**Table** **2**). Figure S1, Supporting Information shows statistics for three identically processed modules type 2. Though stability investigations were outside the scope of this study, Figure S1, Supporting Information also shows shelf stability of the same three modules after more than 6300 h in the glovebox. No sign of degradation is observed.

**Table 2:** Calculated average performance values per sub‐cell within the module type 2 compared to the average measured performance and optical simulations for a single cell with comparable layer thickness and architecture.

	Calculated average performance of cell within module		Average measured performance of single cell (total of 19 cells)	Optical simulations for cell stack
	module area	active area: corrected for *GFF* losses		
*J*_SC_ [mA cm^−2^]	12.8 | 13.9		14.7 ± 0.18	16.1
*V*_OC_ [V]	0.84		0.84	–
*FF* [%]	0.73		0.70	–
PCE [%]	7.9 | 8.6		8.6 ± 0.12	–
AVT [%]	50.8		51.9	52.2
LUE [%]	4.00		4.44 ± 0.06	–

**Figure 6:**
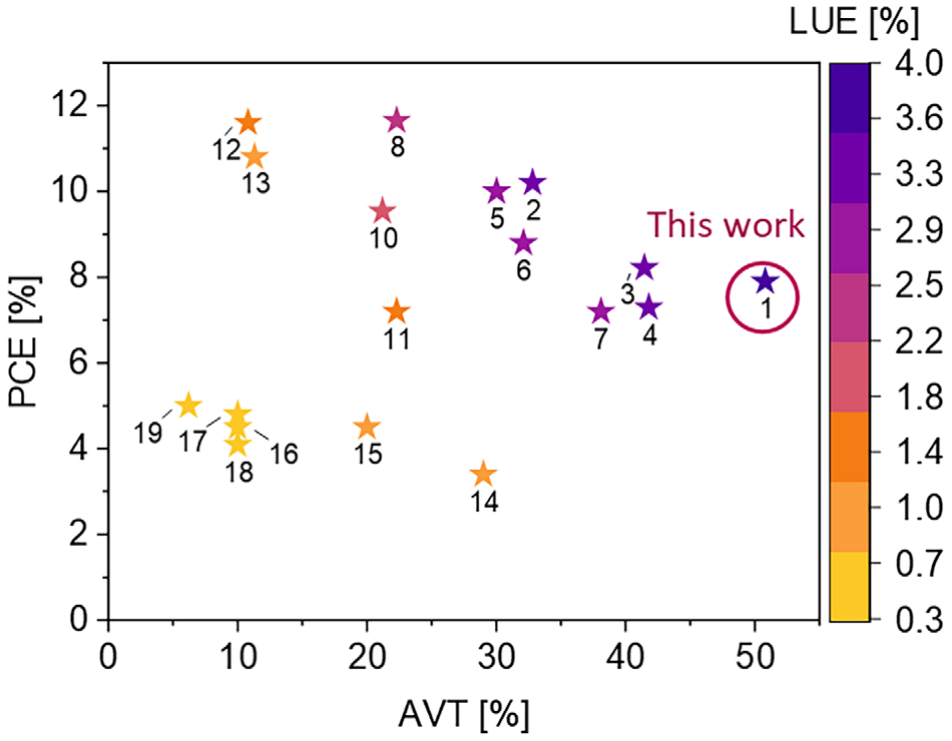
Literature values for PCE, AVT and LUE for STOSM compared to this work. It should be noted that module size is not shown as a separate parameter in this graph, but can be looked up in Table 3.

Similar to the module type 1, losses in the module type 2 compared to the single cell are particularly notable when it comes to *J*_SC_. Both optically simulated and measured single cell data show higher *J*_sc_ values (16.1 and 14.7 mA cm^−2^, respectively) than the module's calculated average *J*_SC_​ per sub‐cell of 12.8 mA cm^−2^, partially attributed to losses in *GFF* introduced by the 24 interconnections within the module. When correcting for those *GFF* losses, the *J*_SC_ increases to 13.9 mA cm^−2^, resulting in a corrected PCE of 8.6% which is equivalent to the single cell PCE of 8.6%. The *V*_OC_​ remained consistent at 0.84 V across both the measured single cell and the module´s sub‐cell average, while the fill factor showed a modest increase in the module (0.73) compared to the single cell (0.70). Additionally, minor fluctuations in material concentration or temperature can significantly impact the position of the transmission maximum and thus cause small variations in AVT. To demonstrate the significance of this work, the PCE, AVT, and LUE of this module are compared to module performance from the literature in **Figure** **6** and **Table** **3**.

**Table 3:** PCE, AVT, LUE, module size, and respective reference data from the literature compared to our work.

Nr.	PCE [%]	AVT [%]	LUE[%]	Module size [cm^2^]	Refs.
1	7.9	50.8	4.0	11.4	Our work
2	10.2	32.8	3.4	21	[11]
3	8.2	41.5	3.4	35	[29]
4	7.3	41.8	3.1	12.8	[30]
5	10.0	30.0	3.0	4.7	[31]
6	8.8	32.1	2.8	4.7	[31]
7	7.2	38.1	2.7	9	[32]
8	11.7	22.3	2.6	18.73	[33]
9	8.2	32.7	2.5	100	[34]
10	9.5	21.2	2.0	10.8	[35]
11	7.2	22.3	1.6	30	[36]
12	11.6	11.0	1.3	18	[37]
13	10.8	11.3	1.2	10.2	[23]
14	3.4	29	1.0	38.71	[24]
15	4.5	20.0	0.9	114.5	[38]
16	4.5	10.0	0.5	216	[39]
17	4.8	10.0	0.5	197.4	[40]
18	4.1	10.0	0.4	216	[39]
19	5.0	6.2	0.3	59.52	[41]

Last, Figure S3, Supporting Information shows the chromaticity diagram (CIE 1931) and the corresponding color rendering index of module type 2.

## 3 Conclusion

In conclusion, two semitransparent organic module designs with an area of 11.4 cm^2^ – solution processed from halogen‐free solvents – are presented. To minimize active area loss to the interconnection of the sub‐cells, precise laser patterning is used. In a new approach, this interconnection is established by a direct connection between the metal‐free top electrode comprising SCA2003 and an Ag layer within the back electrode. By integrating 12 cells in series that incorporate an AZO|Ag|AZO back electrode, initial experiments achieve a power conversion efficiency (PCE) of 6.1% with a high *GFF* of 96.8% and an average visible transmission (AVT) close to that of the single cell. Next, additional SiO₂ and TiO₂ layers were integrated behind the back electrode to enhance optical properties, however first attempts faced challenges with shunting, leading to FFs below 30%. To address these limitations, laser ablation parameters were systematically optimized, changing from a UV picosecond laser to an IR femtosecond laser for the structuring of the PEDOT:PSS top electrode (P3 laser line), and refining the energy and pitch for selective layer ablation. These refinements allowed the precise removal of the top PEDOT:PSS layer without damaging the silver layer in the back electrode, improving electrical contact and therefore module performance. Due to the minimized interconnection loss area, the optimized sub‐cell width was reduced from 2.5 mm to only 1.25 mm. Incorporating a protective sputtering enclosure further enhanced module performance by improving the electrode quality, leading to an increase in PCE to 7.9%, with improved *V*_OC_ and *J*_SC_ and an AVT of 50.8%. These optimization steps collectively resulted in a module with 92% of the single‐cell PCE, more than 98% of the single‐cell AVT, a robust *GFF* of 92.3%, and a record module light utilization efficiency of 4.0%. These results showcase substantial gains in both efficiency and scalability for practical applications in semitransparent photovoltaic systems.

## Conflict of Interest

The authors declare no conflict of interest.

## Supporting information



Supporting Information

## Data Availability

The data that support the findings of this study are available from the corresponding author upon reasonable request.
