# Supplementary material for: Metal‐Less Top Electrode Semitransparent Organic Solar Modules with an Average Visible Transmission of 51% and a Light Utilization Efficiency of 4%
Source: Adv Sci (Weinh). 2025 Jun 23;12(35):e07521. doi: 10.1002/advs.202507521 (PMC12462915; doi:10.1002/advs.202507521)
Supplement: Supplementary file 1 — Supporting Information [file ADVS-12-e07521-s001.pdf]

ADVANCED SCIENCE
Open Access

Supporting Information

for *Adv. Sci.*, DOI 10.1002/advs.202507521

Metal-Less Top Electrode Semitransparent Organic Solar Modules with an Average Visible Transmission of 51% and a Light Utilization Efficiency of 4%

*Leonie Pap, Mathias List, René Haberstroh, Lasse Bienkowski, Martin Mattenheimer, Thomas Kroyer, Jared Faisst, Birger Zimmermann and Uli Würfel**

Supporting Information

Metal-Less Top Electrode Semitransparent Organic Solar Modules with an Average Visible Transmission of 51% and a Light Utilization Efficiency of 4%

*Leonie Pap, Mathias List, René Haberstroh, Lasse Bienkowski, Martin Mattenheimer, Thomas Kroyer, Jared Faisst, Birger Zimmermann, Uli Würfel**

1. **Performance parameters of three modules with the type 2 layer stack: Initial measurement and measurement after 6312 h stored under inert conditions without illumination**

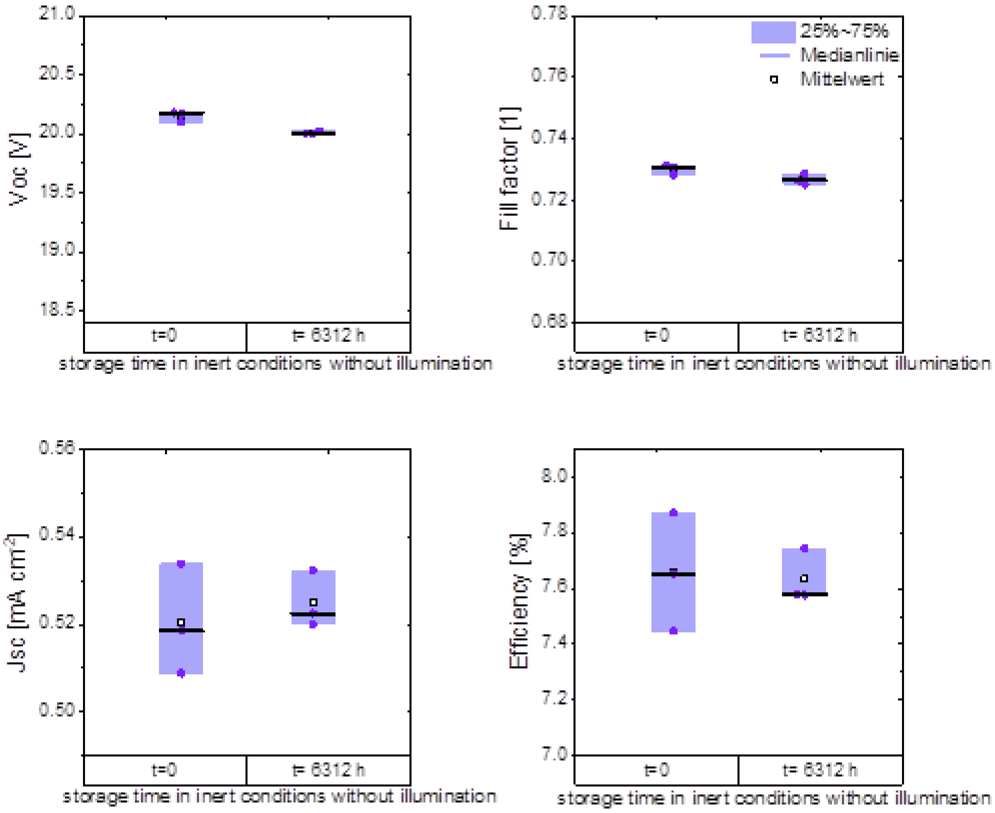

Figure S1. a) V_{OC}, b) *FF*, c) J_{SC}, and d) Efficiency of three modules type 2 once measured initially (t= 0 h) and once after 6312 h stored in the glovebox under inert conditions without illumination.

2. Relative and absolute EQE and APCE of a Module Type 2

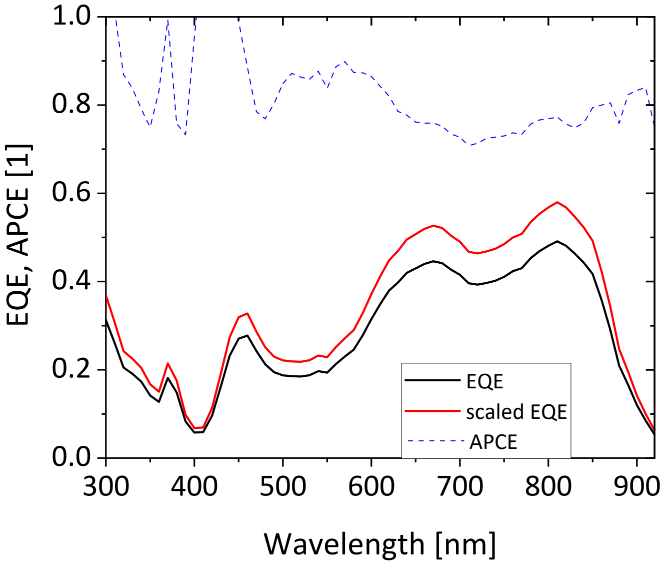

Figure S2. External Quantum Efficiency (EQE) of module type 2. The measured relative EQE (black) was scaled to absolute values (red) by normalizing with the ratio of the measured short-circuit current density to the current density calculated from the relative EQE and the AM1.5G spectrum. The Absorbed Photon-to-Current Efficiency (APCE, blue) was derived by dividing the absolute EQE by the simulated absorption spectrum of the active layer.

The APCE values, apart from larger uncertainties in the wavelength region 300-450 nm, show no pronounced dependence on photon energy.

3. CIE 1931 Chromaticity Diagram and Color Rendering Index of Module Type 2

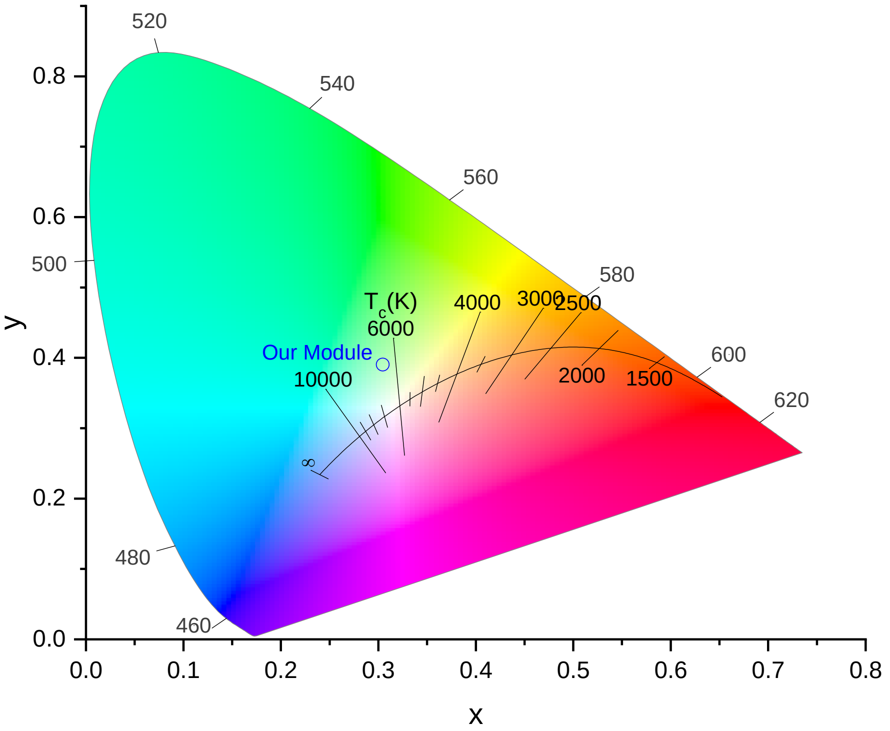

Figure S3. Chromaticity Diagram (CIE 1931) of module type 2 with xy coordinates 0.30444 and 0.39053, and a*,b* (-118.64, 77.79), respectively. The color rendering index (CRI) calculated for the standard D65 is 75.0. When calculating the input Spectral Power Distribution (SPD) using AM1.5G instead of D65, the CRI is 76.8. It should be noted, however, that the CRI is typically used as a figure of merit for white color light sources and has been criticized for its lack of ability to evaluate color quality for different sources accurately.[[1–3]]

4. Experimental Section

Device Fabrication and Characterization

In a first step in the fabrication of semi-transparent organic solar modules 50 × 50 mm^2 glass substrates purchased from Glasmanufaktur Pfähler were cleaned in ultrasonic baths of acetone, isopropanol and water for 10 min each and dry-blown with nitrogen. Alternating layers of TiO_2, SiO_2, AZO, and Ag were sputtered full area onto the glass substrates (AZO|Ag|AZO for the single silver electrode i.e. type 1 and TiO_2|SiO_2|TiO_2|AZO|Ag|AZO for the extended electrode i.e. type 2). A support structure of 4 nm Cr followed by 100 nm Au was deposited via thermal

evaporation at a pressure below 10^{-5} mbar on the top and bottom side of the substrates, outside of the active area. Laser structuring with a UV picosecond laser was used to ablate parts of the bottom electrode until the substrate glass surface (P1) to enable the interconnection of the cells. For the extended back electrode modules, a ZnO solution (N11 from Avantama diluted down to 1% nanoparticles in isopropanol) was spin-cast at 4200 rpm at RT in a nitrogen-filled glove box (GB) and annealed for 10 min at 110 °C. Then PV-X Plus from Raynergy Tek (mixture of PV2300:PV-A-3:N1100 in a ratio of 1:1:0.2 with a concentration of 20 mg mL^{-1}) was spin-cast (GB) from o-xylene at 60 °C and annealed for 10 min at 110 °C. A 40 nm HTL-X layer (purchased from Raynergy Tek) was spin-cast (GB) on top of the photoactive layer and annealed for 2 mins at 110 °C. The P2 laser structuring was performed using an UV picosecond laser, selectively ablating until the silver layer within the bottom electrode. Next, a 90 nm CLEVIOS^{TM} F HC SOLAR (R&D GRADE SCA 2003, supplied by Heraeus) was spin-cast (GB) and annealed for 5 min at 110 °C (Details regarding the CLEVIOS F HC SOLAR material can be found in our work [[4]]). The module with the extended back electrode (type 2) was only annealed in the end for 10 mins at 110 °C instead of annealing each of the layers separately. A P3 laser line was included to complete the interconnection with an IR femtosecond laser, selectively ablating the top SCA2003 layer. Lastly, a P4 laser line was implemented to define the cell stripe length using the UV ps laser. The HTL layers were structured before measuring by taping followed by a photoactive layer structuring by wiping with o-xylene.

Characterization

Current–voltage measurements under simulated AM1.5G light by a class A solar simulator (Newport SP94063A-SR1-167, corrected for spectral mismatch) were carried out with a computer-programmed Keithley 2400 source meter.

UV/VIS measurements were carried out from 280 to 1200 nm by a Perkin Elmer Lambda 950 UV/Vis/NIR spectrophotometer.

Layer thicknesses were determined with a Veeco Dektak 150 measuring samples of single layers on glass substrates.

Suns-V_{OC} Measurement and Pseudo Fill Factor

The intensity dependent open circuit voltage measurements (referred to as Suns-V_{OC}) were conducted directly on the solar simulator (Newport SP94063A-SR1-167). The intensity was adjusted using neutral-density filters and measured using a reference silicon solar cell. Reconstructing a pseudo-J-V curve requires assumptions on the generated current density, which is approximated here by the J_{SC}. Further, it is assumed that the generated current density scales linearly with the intensity I_{illum}.[[5]] The current density can then be approximated by

$$J = J_{SC}\left(\frac{I_{illum}}{I_{illum,1sun}} - 1\right) \tag{2}$$

and plotted versus the measured $V_{OC}(I_{illum})$ in figure 4 c and d. The obtained pseudo J-V curve was fitted with an effective diode equation to obtain the pseudo fill factor.

Optical analysis

Optical modeling was carried out using the transfer matrix method within the CODE/SCOUT software package developed by W. Theiss. To obtain accurate optical coefficients, including the refractive index (n) and extinction coefficient (k) for each material, a Kim oscillator model was used. This model was fitted to measured reflection and transmission data of individual layers with specific thicknesses on a glass substrate. The resulting optical coefficients were then used to simulate the solar cell stack, with defined layer thicknesses, allowing for theoretical calculations of current generation and AVT considering optical interference effects. By adjusting the fit parameters, we optimized layer thicknesses to achieve maximum AVT and current generation.

AVT serves as a key metric in characterizing semitransparent solar cells, integrating the transmission spectrum of the solar cell stack T(λ), the intensity profile of the solar AM1.5 G

spectrum S(λ), and the photopic sensitivity of the human eye P(λ). The AVT is defined as follows:[[6]]

$$\mathrm{AVT} = \frac{\int \mathrm{T}(\lambda)\ \mathrm{P}(\lambda)\ \mathrm{S}(\lambda)}{\int \mathrm{P}(\lambda)\ \mathrm{S}(\lambda)} \tag{3}$$

Module cell stripe optimization

The module optimization was done using a computer program that solves the distributed resistance problem in one dimension.[[7; 8]] In a monolithic series circuitry, the current in the two electrodes is zero far from the circuitry P2 line and adds up the closer the location is to the P2 line. How much current is added at each location is determined by the local voltage V(x) and the JV-curve of the basic infinitesimal cell element. The JV-curve of a real OPV device was fitted with a simple 1-diode model including an R_P:

$$J(V) = a * \exp(b * V) + J_\mathrm{P} + \frac{V}{\rho_\mathrm{P}}, \tag{4}$$

including area normalized JV-fit-parameters as follows:

$a = 10^{-5}\ \mathrm{A/m^2}$

$b = 20\ \mathrm{V^{-1}}$

$J_\mathrm{P} = -170\ \mathrm{A/m^2}$

$\rho_\mathrm{P} = 85\ \Omega\mathrm{m^2}$

This fit does not claim any physical meaning but allows the program to quickly calculate J(V). For ρ_sheet, the sum of both individual sheet resistances of the two electrodes should be used, which in our case is largely dominated by the PEDOT:PSS resistance. The problem is described by the following equations:

$$\frac{dV(x)}{dx} = -\rho_\mathrm{sheet} \cdot I(x), \tag{5}$$

$$\frac{dI(x)}{dx} = -\mathrm{J}(\mathrm{V}(\mathrm{x})), \tag{6}$$

$V(0) = V_0$,

$I(0) = 0$.

I(x) is the curerent flowing in the electrode towards the contact position at the P2 line. At the far end (P3 line, x = 0) the current $I(0)$ is zero. V_0 is varied by the program, and the current and respective Voltage drop are integrated over the width of the cell strip to yield V(P2) and I(P2). By varying V_0, the module JV-curve is reconstructed and the maximum power (P_{MPP}) is calculated. The efficiency is then calculated by dividing the power by the total module period, which is the sum of the active cell strip width and the circuitry loss:

$$PCE_{\mathrm{Module}} = \frac{P_{\mathrm{MPP,Module\ stripe}}}{\mathrm{loss} + \mathrm{width\ of\ active\ cell\ stripe}} \quad (7)$$

The program can also include a series and parallel resistance at the contacts, which we have set to ideal values ($R_S = 0\ \Omega$, $R_P = 5e^{90}\ \Omega$). A non-zero R_S can be caused by contact resistance or incomplete or excessive ablation at the P2 line, while a non-infinite R_P can be caused by defects at the P1 or P3 lines. This sequence is repeated for varying cell strip widths to yield the curves shown in **Fehler! Verweisquelle konnte nicht gefunden werden.**a.

Two-diode model with series and parallel resistance

The equation for the two-diode model is as follows:

$$J(V) = J_{01}\left[\exp\left(\frac{\mathrm{e}_0(V - JR_S)}{\mathrm{k_B}T}\right) - 1\right] + J_{02}\left[\exp\left(\frac{\mathrm{e}_0(V - JR_S)}{2\,\mathrm{k_B}T}\right) - 1\right] + \frac{V - JR_S}{R_P} - J_{\mathrm{Gen}} \quad (8)$$

In order to determine the parameters J_{01} and J_{02}, the 1-sun JV-curve of a very good small area cell was fitted. The following parameters were extracted and used further for the simulations of the light-intensity dependence of the modules (results shown in **Fehler! Verweisquelle konnte nicht gefunden werden.**c,d):

Symbol	Value
J_{01}	2×10^{-14} mAcm^{-2}
J_{02}	10^{-6} mAcm^{-2}
$\mathrm{k_B}T$	0.025851 eV

Statistical Analysis

1. Pre-processing of data: The data was neither transformed nor normalized. For the shown module results, from the measured data shown in the manuscript, no outliers or data points were removed.
2. Data presentation: When applicable, the average values are provided (the sum of all values divided by the number of values) together with the standard deviation (mean ± SD).
3. Sample sizes are provided in the text for each statistical analysis.
4. Software used for statistical analysis was OriginPro 2021.

References

[1] K.A.G. Smet, W.R. Ryckaert, M.R. Pointer, G. Deconinck, P. Hanselaer, A memory colour quality metric for white light sources, 2012, Energy and Buildings, Vol. 49, 216–225,10.1016/j.enbuild.2012.02.008

[2] Kevin A. G. Smet, Wouter R. Ryckaert, Michael R. Pointer, Geert Deconinck, Peter Hanselaer, Memory colours and colour quality evaluation of conventional and solid-state lamps, 2010, Opt. Express, Vol. 18, 26229–26244,10.1364/OE.18.026229

[3] Kevin Smet, Wouter R. Ryckaert, Michael R. Pointer, Geert Deconinck, Peter Hanselaer, Correlation between color quality metric predictions and visual appreciation of light sources, 2011, Opt. Express, Vol. 19, 8151–8166,10.1364/OE.19.008151

[4] Pap, L., Schirmacher, B., Bloch, E., Bogati, S., Viehmann, P., Scheel, A., Müller, D., List, M., Zimmermann, B., Würfel, U., Improved Light Utilization Efficiency for an ITO-Free Semitransparent Organic Solar Cell Using a Multilayer Silver Back Electrode as Infrared Mirror, 2023, Solar RRL, Vol. 7,10.1002/solr.202300561

[5] Schiefer, S., Zimmermann, B., Glunz, S.W., Würfel, U., Applicability of the Suns-Voc Method on Organic Solar Cells, 2014, IEEE J. Photovoltaics, Vol. 4, 271–277,10.1109/JPHOTOV.2013.2288527

[6] Chenchen Yang, Dianyi Liu, Matthew Bates, Miles C. Barr, Richard R. Lunt, How to Accurately Report Transparent Solar Cells, 2019, Joule, Vol. 3, 1803–1809,10.1016/j.joule.2019.06.005

[7] Markus Glatthaar, Zur Funktionsweise organischer Solarzellen auf der Basis interpenetrierender Donator/Akzeptor-Netzwerke, PhD thesis, University of Freiburg, 2007

[8] Alexis de Vos, The distributed series resistance problem in solar cells, 1984, Solar Cells, Vol. 12, 311–327,10.1016/0379-6787(84)90110-8
